# Exploring the clinical application value of peripheral blood T lymphocyte subset in patients with asymptomatic omicron infection

**DOI:** 10.1186/s40001-023-01187-3

**Published:** 2023-07-05

**Authors:** Tuantuan Li, Jing Xu, Yong Gao, XiaoWu Wang, Yuanhong Xu

**Affiliations:** 1 Department of Clinical Laboratory, First Affiliated Hospital of Anhui Medical University, No. 218 of JiXi Road Hefei, Hefei, 230022 Anhui Province China; 2 Department of Clinical Laboratory, The Second People’s Hospital of Fuyang City (Fuyang Infectious Disease Clinical College of Anhui Medical University), Fuyang, 236000 Anhui Province China; 3 Department of Clinical Laboratory, Fuyang People’s Hospital (Anhui Medical University, Affiliated Fuyang Peoples Hospital), Fuyang, 236000 Anhui Province China

**Keywords:** Coronavirus disease 2019, OMICRON variant strains, CD3CD4+, T lymphocytes

## Abstract

**Objective:**

To investigate the clinical significance and value of peripheral blood T lymphocyte subset in patients with asymptomatic novel coronavirus variant strains infection (OMICRON).

**Methods:**

A retrospective analysis of 281 patients with asymptomatic OMICRON infection who were admitted and isolated to the Fuyang Second People's Hospital from March to April 2022 was conducted. With 32 normal people as the control group, T lymphocytes of the two groups (CD3 + T, CD3 + CD4 + T, CD3 + CD8 + T) were analyzed and the differences between the two groups were analyzed. CD4 + T lymphocytes between patients with asymptomatic OMICRON infection and patients with mild COVID-19 infection in 2020 were analyzed and compared. Based on CD3 CD4 + T lymphocyte changes, lymphocyte reference range: CD3 CD4 + T lymphocyte count 404–1612/μL. Lower than 404 × 106/μL was defined as lymphocytopenia, patients were divided into the reduced group (138) and the normal group (143). The CT value of novel coronavirus nucleic acid (ORF1ab gene, N gene) and the time of viral shedding were compared between the two groups.

**Results:**

Differences in number of CD3 + T cells, CD3 + CD4 + T cells, and CD3 + CD8 + T cells were significant between both groups (*P* < 0.05), which were significantly higher in the normal population than in the patients with asymptomatic OMICRON infection. There was no significant difference in CD4 + T lymphocytes between patients with asymptomatic OMICRON infection and patients with mild COVID-19 infection in 2020 (*P* < 0.05). The novel coronavirus nucleic CT value was significantly lower in the CD3CD4 + T lymphocyte-reduced group than in the CD3CD4 + T lymphocyte-normal group (*P* < 0.05). Moreover, the time of viral shedding was significantly longer in the reduced group compared with the normal group (*P* < 0.05).

**Conclusion:**

The changing characteristics of the peripheral blood T lymphocyte subset count in patients with asymptomatic OMICRON infections can provide an important basis for the diagnosis and outcome of the asymptomatic OMICRON infection.

## Introduction

Coronavirus disease 2019 (COVID-19), as a new acute respiratory infectious disease, has been reported hundreds of millions of infections worldwide, becoming a major global public health event [1]. Coronavirus is a large group of viruses widely found in nature, and COVID-19 belongs to the β coronavirus [2]. The Omicron variant has up to 36 substitutions in the viral spike protein, and 59 mutations across the genome. Omicron was found to circumvent the neutralizing of infection and vaccine-induced antibodies [3, 4]. COVID-19 is known to attack people's immune system. Shortened lymphocyte half-life, increased apoptosis, and increased adrenal corticoid secretion led to a significant reduction in peripheral blood T lymphocytes and subsets [5]. In just more than 2 years, the virus has experienced many mutations. The Omicron variant strains found in South Africa are highly insidious and spreading rapidly, making the outbreak situation appear again in many countries and regions, and the number of infections and deaths remains high, leading to a new wave of novel coronavirus pandemic [6]. The emergence of the Omicron is another blow to the already troubled global economy, and will certainly add more uncertainty to all of us around the world.

Patients infected with SARS COV2 generally experience three immune response processes, namely innate immune response, humoral immune response and cellular immune response, plus cytokine storm. Innate immunity is the first line of defense against the invasion of SARS-COV-2, in which neutrophils and mononuclear macrophages play an important role. In the process of clinical treatment, we should pay attention to their changes and timely control and intervene the development of immune response. In addition, SARS-CoV-2 mainly uses cellular immunity mediated by Th1 cells and CTL cells to control virus infection. Neutralizing antibodies produced by B cells in humoral immunity can prevent the combination of virus and host cells and reduce the invasion of virus to host cells to play a protective role. Humoral immunity is mainly mediated by antibodies produced after B cell activation. Studies have shown that T cells are important cells for assisting humoral immunity, directly killing and clearing SARS-CoV-2. It is necessary to pay close attention to T cells in clinical diagnosis and treatment, especially the changes in the number and function of CD4 + and CD8 + T cells, which will directly affect the choice of late treatment. The increase in the number of T cells is regarded as a key indicator for the good prognosis of COVID-19 patients, and adopt-specific T cells are helpful for the recovery of patients [7]. When the virus variant is different, the main immune modes still exist, but with the emergence of the new subtype of virus variant, the virus will appear higher infectivity, stronger vaccine breakthrough ability and severe antibody escape rate in the immune response [8]. There are some differences in the immune response with different virus variants. In general, the virus variants are different, and the main types of immunity still exist, namely innate immunity, humoral immunity, and cellular immunity. However, with the emergence of new subtypes of virus variants, the internal response methods of the three immune responses will be different, such as changes in the response receptors, higher infectivity of the virus during the immune response, stronger vaccine breakthrough ability, and severe antibody escape rate. In infection with other SARS-CoV2 variants, differences in the immune response to parts of LT occur. For example, in a new study, researchers from Imperial College London and Queen Mary University of London found that the first SARS-CoV-2 spike protein that people encounter through vaccination or infection affects their subsequent immune responses to current and future SARS-CoV-2 variants. That is, the different properties it confers have an impact on the ability of the immune system to protect against the SARS-CoV-2 variant, and also on the rate at which that protection decays. Each SARS-CoV-2 variant has a different mutation in the spike protein, and these authors found that these mutations shape the subsequent antibody and T-cell response (immune repertoire) [9].

The Huazhong University of Science and Technology team was the first to complete the pathologic autopsy of the remains of nine COVID-19 victims. They found that the virus damages not only the lungs of infected people, but also the immune system and other tissues and organs. They found that WBC count, Neu count, Lym count and neutrophil–lymphocyte ratio (NLR) value in peripheral blood cell detection can be used to assist in the differentiation of COVID-19 patients, H1N1 patients and healthy people, which has certain clinical significance for the diagnosis of COVID-19 [10].

We performed a retrospective analysis of peripheral blood lymphocytes from 281 patients with asymptomatic OMICRON variant strains infection admitted to Fuyang Second People's Hospital, to explore the correlation analysis of peripheral blood lymphocyte subsets and asymptomatic infection of novel coronavirus OMICRON variant strains, to find a good biological indicators to predict early disease classification and evaluate disease outcome and improve the clinical diagnosis of novel coronavirus OMICRON variant strains infection.

In addition to the strong infectivity and transmission of the novel coronavirus, the global "pandemic" and spread of the novel coronavirus are closely related to the flow of people and economic and trade exchanges among regions. In addition to the strong infectivity of the novel coronavirus itself at the beginning of the epidemic, the second is the close population movement between the epicenter of the epidemic. As well as a number of demographic and sociological characteristics will affect the incidence of COVID-19 infection. Therefore, subjects were included and excluded according to the following general demographic characteristics: gender, age, Marital status, Level of education, professional, The household registration, Total Covid-19 knowledge score, Health literacy of infectious disease prevention and control, Sense of self anxiety, Self-reported health, Chronology of chronic diseases, Experience of the covid-19, Blood Group (GWAS Gene Big Data Analysis), and so on.

## Objects and methods

### Case selection

This study was retrospective. Data of patients with asymptomatic infection of novel coronavirus OMICRON variant strains admitted and isolated in Fuyang Second People's Hospital from March 2022 to April 2022 were collected for study. According to the National Health Commission's COVID-19 Diagnosis and Treatment Program (Trial Ninth Edition), these patients were diagnosed as COVID-19 and confirmed as the OMICRON variant strains by genotyping [11]. In 2020, 54 patients were mildly infected with the novel coronavirus. Inclusion criteria: (1) confirmed as B.1.1. 529 (OMICRON) variant strains by genome sequencing; (2) The patients had chest CT examinations for 3 or more times. Exclusion criteria: (1) Patients complicated with other pulmonary infections, lung metastases, and pneumoconiosis; (2) Patients with less than 3 nucleic acid tests. Based on the presence or absence of pneumonia changes, 281 asymptomatic infection patients were confirmed. The study protocol has been reviewed and approved by the Ethics Committee of Fuyang Second People's Hospital.

### 1.2 Research methods

Patients were grouped according to the routine blood results after admission. Patients with asymptomatic OMICRON infection were classified into the CD3CD4 + T lymphocyte-reduced group (138) and the CD3CD4 + T lymphocyte-normal group (143) (Control group). Lymphocyte reference range: CD3CD4 + T lymphocyte count 404 ~ 1612/μL. Less than 404 × 106/μL was defined as lymphopenia.

### Instruments and reagents

Lymphocytes: 5 mL peripheral blood was collected and added into a serum anticoagulant tube, mixed thoroughly and sent to the clinical laboratory at low temperature. The absolute counts of CD3 + and CD4 + T lymphocyte subsets were detected by flow cytometry [BriCyte E6 flow cytometry (Mindray)]. The reagents were provided by Shenzhen Mindray Bio-Medical Electronics Co., Ltd., and operated in strict accordance with the instructions.

Nucleic acid CT value detection: According to the Technical Guidelines for Laboratory Detection of Pneumonia Infected by Novel Corona Virus and the instructions for use of the kit, throat swabs and sputum samples were collected from patients and stored separately in a special sample bank. Viral nucleic acid was extracted using nucleic acid extraction kit from Beijing Jinhao Pharmaceutical Co., Ltd. Nucleic acid was detected using 2019-nCov nucleic acid detection kit for novel coronavirus from Shanghai Bergey Medical Technology Co., Ltd., and the operation and result judgment were performed in strict accordance with the kit instructions. Sample processing, nucleic acid extraction and detection are performed in the secondary biosafety laboratory by the personnel who have received relevant technical safety training in strict accordance with relevant guidelines and instructions for use of the kit.

### Statistical methods

Statistical data analysis was performed using the SPSS 23.0 software. Measurement data were tested for normality using the Kruskal–Wallis test. Means ± standard deviations (${\overline{\text{X}}}$ ± S) were used for measurement data that obeyed normal distribution, and medians and interquartile ranges were used for statistical description of measurement data that did not obey normal distribution. Comparisons between groups were performed using Student-*t* test or Mann–Whitney *U* test. Enumeration data were statistically described using number of cases (%). Chi-square test was used for comparison between groups. *P* < 0.05 indicates a statistically significant difference.

## Results

### Patient demographics and clinical information

There were 138 patients in the lymphopenia group, 85 males, mean age 17.67 years, first NLR 4.65, length of hospital stay 16.83, and duration of infection 14.83; 143 patients in the lymphopenia group, 82 males, mean age 25.63 years, first NLR 1.61, length of hospital stay 14.10, and duration of infection 12.10. Patients in both groups had no underlying chronic diseases and survived after treatment. Age and sex were not significantly different between groups (see Table 1).

**Table 1:** Demographic characteristics and clinical information of OMICRON asymptomatic infected patients

Study group (*n*)	Gender (M/F)	Age (years)	Presence or absence of chronic underlying disease (yes/no)	First NLR	Length of stay (days)	Outcome (death/survival)	Duration of infection (days)	Blood sampling period
CD3CD4 + T lymphocyte-reduced group (*n* = 138)	85/53	17.67 ± 13.18	No	4.65 ± 4	16.83 ± 7.26	Survival	14.83 ± 7.26	April 1–April 14, 2022
Control group (*n* = 143)	82/61	25.63 ± 12.66	No	1.61 ± 0.94	14.10 ± 6.38	Survival	12.10 ± 6.38	April 1–April 15, 2022
*X*^2^/*t* value	0.526	0.171	–	8.841	3.349	–	3.349	–
*P* value	0.468	0.864	–	< 0.001	< 0.001	–	< 0.001	–

*X*^*2*^ value is the test statistic for performing chi-square test, *t* value is the test statistic for performing *t*-test. The *P* value is the probability that (*F* test or *T* or any other test) is greater than the desired value. *P* < 0.05 indicates statistical significance

*NLR* neutrophil–lymphocyte ratio

There were a total of 54 patients with mild COVID-19 infection in 2020, 31 males and 23 females, with a mean age of 40.45, a first NLR of 3.56, a mean length of hospital stay of 20.61, and a mean duration of infection of 17.61. All patients had no underlying chronic disease and survived treatment. Gender and first NLR were not significantly different compared to patients with asymptomatic OMICRON infection (see Table 2).

**Table 2:** Basic information for younger patients 2020

Study group (*n*)	Gender (M/F)	Age (years)	Presence or absence of chronic underlying disease (yes/no)	First NLR	Length of stay (days)	Outcome (death/survival)	Duration of infection (days)	Blood sampling period
Patients with asymptomatic OMICRON infection (*n* = 281)	167/114	22.40 ± 12.87	No	3.13 ± 2.47	15.47 ± 6.82	Survival	13.42 ± 6.82	April 1–April 15, 2020
Patients with mild COVID-19 infection in 2020 (*n* = 54)	31/23	40.45 ± 15.34	No	3.56 ± 1.93	20.61 ± 7.80	Survival	17.61 ± 7.80	April 1–April 14, 2022
X2/*t* value	0.077	− 9.1	–	− 1.002	-4.894	–	− 3.946	–
*P* value	0.782	< 0.001	–	0.317	< 0.001	–	< 0.001	–

*X*^*2*^ value is the test statistic for performing chi-square test, *t* value is the test statistic for performing *t*-test. The *P* value is the probability that (*F* test or *T* or any other test) is greater than the desired value. *P* < 0.05 indicates statistical significance

### Comparison between patients with asymptomatic OMICRON infection and the normal population

There were differences in peripheral blood CD3 + T, CD3 + CD4 + T, and CD3 + CD8 + T lymphocyte count of the first blood routine after admission between the 281 patients with asymptomatic OMICRON infection and the normal population. The number of CD3 + T, CD3 + CD4 + T, and CD3 + CD8 + T cells was significantly reduced in the asymptomatic infected individuals with OMICRON, and the difference was statistically significant (*P* < 0.05). Among them, CD3 + T lymphocytes decreased most significantly (*z* =  − 6.191, *P* < 0.05) (see Table 3).

**Table 3:** Comparison of CD4 + T lymphocytes of the first time after admission between OMICRON asymptomatic infected patients and the normal population

Study group (*n*)	level of LT CD3 +	Level of LT CD3 + CD4 +	Level of LT CD3 + CD8 +
Patients not infected with the Omicron variant (*n* = 32)	1298 (1080–1494)	626 (566–781)	538 (394–704)
Patients with asymptomatic OMICRON infection (*n* = 281)	795 (520–1046)	407 (266–572)	304 (198–416)
*Z* value	− 6.191	− 5.546	− 5.793
*P* value	< 0.001	< 0.001	< 0.001

*Z* value is the test statistic for performing rank sum test; The *P* value is the probability that (*F* test or *T* or any other test) is greater than the desired value. *P* < 0.05 indicates statistical significance

### Comparison between patients with asymptomatic OMICRON infection and patients with mild COVID-19 infection in 2020

Peripheral blood CD4 + T lymphocytes in patients with asymptomatic OMICRON infection showed no significant changes compared with that in patients with mild COVID-19 infection in 2020, and the difference was not statistically significant (*P* > 0.05) (see Table 4).

**Table 4:** Comparison of CD4 + T lymphocytes in peripheral blood of asymptomatic OMICRON infected patients and 2020 mild COVID-19 infected patients sampled

Study group (*n*)	LT CD3 + T lymphocyte (count/μL)	LT CD4 + T lymphocyte (count/μL)	LT CD4 + T lymphocyte (percentage, %)
Patients with asymptomatic OMICRON infection (*n* = 281)	830.3. ± 404.7	445.5 ± 230.3	35.9 ± 7.8
Patients with mild COVID-19 infection in 2020 (*n* = 54)	760.3 ± 309.9	401.9 ± 165.6	37.0 ± 8.4
*t* value	1.308	1.766	− 1.061
*P* value	0.192	0.080	0.289

The *t* value is the test statistic for performing the *t*-test; The *P* value is the probability that (*F* test or *T* or any other test) is greater than the desired value. *P* < 0.05 indicates statistical significance

### Comparison between CD3CD4 + T lymphocyte-reduced group and CD3CD4 + T lymphocyte-normal group

The CT value of novel coronavirus nucleic acid at admission was compared between the CD3CD4 + T lymphocyte-reduced group and CD3CD4 + T lymphocyte-normal group. Compared with the CD3CD4 + T lymphocyte-normal group, the lowest circulating threshold Ct value of nucleic acid detection was significantly lower in the CD3CD4 + T lymphocyte-reduced group, and the difference was statistically significant (*P* < 0.05) (see Table 5).

**Table 5:** Comparison of CT values of novel coronavirus at admission between CD3CD4 + T lymphocyte-reduced group and CD3CD4 + T lymphocyte-normal group

Study group (*n*)	ORF1ab gene (CT value)	*N* gene (CT value)
CD3CD4 + T lymphocyte-reduced group (*n* = 138)	24.8 (22.3–28.4)	25.6 (23.2–28.9)
Control group (*n* = 143)	27.9 (24.4–32.5)	28.6 (25.3–32.1)
*Z* value	2.420	2.311
*P* value	< 0.001	< 0.001

*Z* value is the test statistic for performing rank sum test; the *P* value is the probability that (*F* test or *T* or any other test) is greater than the desired value. *P* < 0.05 indicates statistical significance

The time of viral shedding was compared between CD3CD4 + T lymphocyte-reduced group and CD3CD4 + T lymphocyte-normal group. Statistics show that the average time of viral shedding was 14 days in the CD3CD4 + T lymphocyte-reduced group, and 10 days in the CD3CD4 + T lymphocyte-normal group. Patients with decreased CD3 + CD4 + T lymphocytes showed long viral negative periods of time and showed significant statistical differences (*P* < 0.05). See Table 5. The time of viral shedding was longer in the CD3CD4 + T lymphocyte-reduced group, and the difference was statistically significant (*P* < 0.05) (see Table 6).

**Table 6:** Correlation between abnormal *T* lymphocyte number of the first time after admission and time of viral shedding in patients with asymptomatic OMICRON infection

Study group (*n*)	Time of OMICRON viral shedding
LT CD3CD4 + T lymphocyte-reduced group (*n* = 138)	14 (8–21)
Control group (*n* = 143)	10 (7–16)
*Z* value	3.251
*P* value	0.001

*Z* value is the test statistic for performing rank sum test; the *P* value is the probability that (*F* test or *T* or any other test) is greater than the desired value. *P* < 0.05 indicates statistical significance

## Discussion

It was found that in individuals previously infected with Omicron and in vaccinated individuals, they had robust and more T cell responses, with a generally mild disease course [12]. After vaccination, early induction of antigen-specific CD4 + T cells is associated with coordinated generation of antibody and CD8 + T cell responses [13]. Previous studies have also shown that CD8 + T cells play a critical role in alleviating COVID-19 disease severity, inducing long-term immune protection, and CD8 + T cell-mediated rapid viral clearance [14]. Thus whether T cell responses can enhance protection against Omicron infection and disease is quite important for predicting the progression of future viral variants.

The results of this study showed that T lymphocytes and their subsets CD3 + , CD4 + , and CD8 + T cells were significantly reduced in patients with asymptomatic OMICRON infections compared with the normal population, which are consistent with previous studies [15]. It may be that the virus has attacked immune organs such as lymph nodes, causing damage to T lymphocytes and subsets. We next compared peripheral blood CD4 + T lymphocytes between patients with asymptomatic OMICRON infection and patients with mild COVID-19 infection in 2020, and results showed no statistically significant difference. This results confirmed that asymptomatic OMICRON infection, similar to COVID-19 infection, both caused T lymphocytes and subsets damage [16].

Omicron shares the mutant with the alpha, beta, gamma, and delta variants, and other variants of unknown significance [17]. The characteristics of some of these mutations are still under interpretation. Within several months of its appearance, however, it had been split into two lines (BA.1 and BA.2) [18]. In addition, unlike the SARS-CoV-2 virus, omicron is difficult to grow in VERO cells (genetically engineered African green monkey kidney cells), and it is unlikely to cause significant damage (plaque) in these cells [19]. Researchers from Hong Kong reported that omicron grew tenfold faster in the bronchi (considering rapid transmission) than the delta, but 10-times slower in the lungs [20]^.^ The related findings were influenced by the duration of infection with the Omicron variant. Patients infected with the Omicron variant have a longer duration of positive nucleic acid tests than those infected with the common novel coronavirus [21]. Although it is still too early, these observations may suggest that Omicron has less severe disease. Preliminary information on omicron suggests that the median latency of omicron is shorter at about 2–3 days, compared to the 5-day median latency of the original virus [22]. However, these are early impressions and the situation may change over time. It has now also been shown that the SARS-CoV-2-specific T cell responses are critical for the viral clearance [23]. Therefore, in this experiment, we divided them into CD3CD4 + T lymphocytes-reduced group and CD3CD4 + T lymphocyte-normal group according to the CD3CD4 + T lymphocyte changes. The Ct value detected by the real-time fluorescence reverse transcription polymerase chain reaction (RT-PCR) nucleic acid test indirectly reflects the viral load in the sample. The results showed that the Ct value of the CD3CD4 + T lymphocyte-reduced group was significantly lower than the normal group, that is, the CD3CD4 + T lymphocyte-reduced group had high novel coronavirus content. Previous results showed significantly higher viral load in severe COVID-19 patients compared with mild patients. Our results are consistent with that [24]. At the same time, we examined the time of viral shedding in these two groups, and the results showed that the patients in the CD3CD4 + T lymphocyte-reduced group obviously needed a longer time to return to normal. Therefore, CD3CD4 + T lymphocyte changes during omicron infection have some reference significance.

The present study has several limitations: first, the relatively small number of cases. Given the previous reports of more severe infection symptoms in older COVID-19 patients, a larger sample size is needed to assess the disease severity in older and young patients [25]. Second, the lung changes caused by asymptomatic infection of OMICRON variant strains still need further detection and follow-up.

The LT detection method for early diagnosis of COVID-19 mentioned in this paper may not be the only and optimal diagnostic method with the passage of time, and the diagnosis may not be very accurate only by relying on peripheral blood analysis. At the same time, the sample size of this paper may not be large enough. These are the limitations of our research. In future research, we will further expand the sample size, increase the sample representativeness, and select more parameters with better accuracy for research.

## Conclusion

Peripheral blood T lymphocyte subsets of asymptomatic OMICRON infected persons are reduced compared with the normal population, and those with reduced CD3CD4 + T lymphocytes have high novel coronavirus content, which requires longer time of viral shedding. Therefore, the study of peripheral blood lymphocytes changes in patients with asymptomatic OMICRON infection can provide new ideas for the early diagnosis and later treatment of this disease.

## Data Availability

The datasets used or analyzed during the current study are available from the corresponding author on reasonable request.
